# Loss of *Akap1* Exacerbates Pressure Overload-Induced Cardiac Hypertrophy and Heart Failure

**DOI:** 10.3389/fphys.2018.00558

**Published:** 2018-05-28

**Authors:** Gabriele G. Schiattarella, Nicola Boccella, Roberta Paolillo, Fabio Cattaneo, Valentina Trimarco, Anna Franzone, Stefania D’Apice, Giuseppe Giugliano, Laura Rinaldi, Domenica Borzacchiello, Alessandra Gentile, Assunta Lombardi, Antonio Feliciello, Giovanni Esposito, Cinzia Perrino

**Affiliations:** ^1^Department of Advanced Biomedical Sciences, University of Naples Federico II, Naples, Italy; ^2^Department of Molecular Medicine and Medical Biotechnologies, University of Naples Federico II, Naples, Italy; ^3^Department of Neuroscience, Reproductive Science and Odontostomatology, University of Naples Federico II, Naples, Italy; ^4^Department of Cardiology, Inselspital, Universitätsspital Bern, Bern, Switzerland; ^5^Department of Biology, University of Naples Federico II, Naples, Italy

**Keywords:** heart failure, Akt, cardiac hypertrophy, cardiomyocytes, pressure overload

## Abstract

Left ventricular hypertrophy (LVH) is a major contributor to the development of heart failure (HF). Alterations in cyclic adenosine monophosphate (cAMP)-dependent signaling pathways participate in cardiomyocyte hypertrophy and mitochondrial dysfunction occurring in LVH and HF. cAMP signals are received and integrated by a family of cAMP-dependent protein kinase A (PKA) anchor proteins (AKAPs), tethering PKA to discrete cellular locations. AKAPs encoded by the *Akap1* gene (mitoAKAPs) promote PKA mitochondrial targeting, regulating mitochondrial structure and function, reactive oxygen species production, and cell survival. To determine the role of mitoAKAPs in LVH development, in the present investigation, mice with global genetic deletion of *Akap1* (*Akap1*^-/-^), *Akap1* heterozygous (*Akap1*^+/-^), and their wild-type (*wt*) littermates underwent transverse aortic constriction (TAC) or SHAM procedure for 1 week. In *wt* mice, pressure overload induced the downregulation of AKAP121, the major cardiac mitoAKAP. Compared to *wt, Akap1*^-/-^ mice did not display basal alterations in cardiac structure or function and cardiomyocyte size or fibrosis. However, loss of *Akap1* exacerbated LVH and cardiomyocyte hypertrophy induced by pressure overload and accelerated the progression toward HF in TAC mice, and these changes were not observed upon prevention of AKAP121 degradation in seven *in absentia* homolog 2 (*Siah2*) knockout mice (*Siah2*^-/-^). Loss of *Akap1* was also associated to a significant increase in cardiac apoptosis as well as lack of activation of Akt signaling after pressure overload. Taken together, these results demonstrate that *in vivo* genetic deletion of *Akap1* enhances LVH development and accelerates pressure overload-induced cardiac dysfunction, pointing at *Akap1* as a novel repressor of pathological LVH. These results confirm and extend the important role of mitoAKAPs in cardiac response to stress.

## Introduction

Pathological left ventricular hypertrophy (LVH) is a hallmark feature of a number cardiovascular diseases (Hill and Olson, 2008; Schiattarella and Hill, 2015; Schiattarella et al., 2017) and is strongly associated with increased risk of developing heart failure (HF; Levy et al., 1990). In response to stress, such as hypertension and pressure overload, several cellular and sub-cellular modifications lead to cardiac remodeling (Perrino and Rockman, 2007; Burchfield et al., 2013). Despite the critical role of LVH in the development of cardiac dysfunction, the mechanisms underlying cardiomyocyte hypertrophy in response to pressure overload still remain not completely understood.

Members of a family of cyclic adenosine monophosphate (cAMP)-dependent protein kinase A (PKA) anchor proteins (AKAPs) have been identified as potential regulators of cardiac responses to pathological stimuli including pressure overload-induced LVH (Perrino et al., 2010; Diviani et al., 2011; Rababa’h et al., 2014). By anchoring PKA to membranes and cellular organelles, AKAPs play a key role in the intracellular propagation of cAMP/PKA signals (Feliciello et al., 2001; Carlucci et al., 2008b). Several AKAPs are expressed in cardiomyocytes, regulating crucial cellular functions (Diviani et al., 2011). Among these, mAKAP has been shown to regulate hypertrophy of neonatal cardiomyocytes through mitogen-activated protein kinases (MAPKs) signaling pathways (Rababa’h et al., 2014). Moreover, AKAP-Lbc also regulates cardiomyocytes hypertrophy forming a complex with other protein kinases (Carnegie et al., 2008).

Mitochondria are the major energy source for contraction and relaxation in cardiomyocytes. LVH and ultimately HF are characterized by mitochondrial dysfunction leading to reduced ATP production and increased generation of mitochondrial reactive oxygen species (ROS; Torrealba et al., 2017). A sub-family of AKAPs deriving from the alternative splicing of the *Akap1* gene amplifies signals to mitochondria (mitoAKAPs), and has been shown to be critical under several pathological conditions (Carlucci et al., 2008a; Perrino et al., 2010; Scorziello et al., 2013; Schiattarella et al., 2016). We have previously demonstrated that the absence of *Akap1* exacerbates cardiac injury following myocardial infarction in mice, promoting mitochondrial dysfunction, enhancing ROS production and infarct size, and ultimately reducing survival (Schiattarella et al., 2016). Using a rat model of LVH, we have shown that degradation of AKAP121, the most abundant *Akap1* product in muscle cells, occurs early in response to pressure overload, and is associated with impaired mitochondrial function and reduced cell survival (Perrino et al., 2010). Degradation of AKAP121 is mediated, in part, by the E3 ubiquitin ligase seven *in absentia* homolog 2 (*Siah2*; Carlucci et al., 2008a). Previous studies in our laboratory and others have also shown that AKAP121 degradation upon ischemia is reduced in *Siah2* knockout mice (*Siah2*^-/-^; Kim et al., 2011; Schiattarella et al., 2016), and that *Siah2* deletion reduces cardiac susceptibility to ischemia due to loss of *Akap1* (Schiattarella et al., 2016). Although the potential role of AKAP121 in the hypertrophic growth of cardiomyocytes has been suggested by *in vitro* studies (Abrenica et al., 2009), basal cardiac mass, structure, and function of *Akap1*^-/-^ mice were not significantly different compared to their *wt* littermates. Thus, whether *Akap1* plays a causal role during the development of LVH *in vivo* remains unknown. In the present investigation, we hypothesized that loss of *Akap1* exacerbates cardiac hypertrophy in response to pressure overload, leading to an accelerated progression toward HF.

## Materials and Methods

### Experimental Animals

All experiments involving animals in this study were conformed to the Guide for the Care and Use of Laboratory Animals published by the US National Institutes of Health (NIH Publication 8th edition, update 2011), and were approved by the animal welfare regulation of University of Naples Federico II, Naples, Italy, and by the Ministry of Health, Italy. *Akap*1 knockout mice (*Akap*1^-/-^, C57BL/6 background) and *Akap1* heterozygous mice (*Akap*^+/-^, C57BL/6 background) were kindly donated by McKnight G. S. and have been previously described (Newhall et al., 2006; Schiattarella et al., 2016). *Siah2* knockout mice (*Siah2*^-/-^, C57BL/6 background) were kindly donated by Bowtell D. Wild-type (*wt*, C57BL/6 background) *Akap*1^+/-^ and *Akap*1^-/-^ mice of either gender (8–9 weeks old) were included in the study and maintained under identical conditions of temperature (21 ± 1°C), humidity (60 ± 5%), and light/dark cycle, and had free access to normal mouse chow.

### Mouse Model of Pressure Overload-Induced Cardiac Hypertrophy and Heart Failure

Pressure overload was induced in adult *Akap1*^+/-^, *Akap*1^-/-^, and *Siah2*^-/-^ mice and their *wt* littermates by transverse aortic constriction (TAC) as previously described (Esposito et al., 2011; Angrisano et al., 2014). Briefly, mice were anesthetized by intraperitoneal injection of 0.1 ml/kg of mixture of 50% Tiletamine and 50% Zolazepam (Zoletil 100) and Xylazine 5 mg/kg (Sigma-Aldrich) and a surgical suture was passed across the aortic arch between left common carotid artery and innominate artery. Another group of animals underwent a left thoracotomy without aortic constriction (sham). Mice from all the groups were sacrificed 1 week (1w) after surgery to perform molecular analyses. Only TAC animals with systolic pressure gradients >40 mmHg were included in the study.

### Transthoracic Echocardiography

Cardiac function was non-invasively monitored by transthoracic echocardiography using the Vevo 770 high-resolution imaging system equipped with a 30-MHz RMV-707B scanning head (Visual-Sonics, Toronto, ON, Canada) 1w after sham or TAC operation in mice of all genotypes as previously described (Esposito et al., 2011; Perrino et al., 2013).

### Protein Extraction and Immunoblotting

Heart and cellular samples were homogenized in a buffer containing 150 mmol/L NaCl, 50 mmol/L Tris-HCl (pH 8.5), 2 mmol/L EDTA, 1% v/v NP-40, 0.5% w/v deoxycholate, 10 mmol/L NaF, 10 mM sodium pyrophosphate, 2 mmol/L PMSF, 2 heart leupeptin, 2 heart aprotinin, pH 7.4, using the program Protein_1 on a GentleMACS tissue Dissociator (Miltenyi Biotec; Esposito et al., 2015; Cattaneo et al., 2016). Protein concentration in all lysates was measured by using a dye-binding protein assay kit (Bio-Rad) and a SmartSpec Plus spectrophotometer (Bio-Rad) reading at a wavelength of 595 nm. Immunoblotting was performed by using commercially available antibodies: anti-Akt (rabbit polyclonal, Santa Cruz Biotechnology), anti-pAkt (Ser473, rabbit polyclonal, Cell Signaling), anti-cleaved Caspase-3 (rabbit polyclonal, Cell Signaling), anti-AKAP121 (Carlucci et al., 2008a; rabbit polyclonal), anti-caspase-9 (rabbit polyclonal, Santa Cruz Biotechnology), anti-IDH2 (goat polyclonal, Santa Cruz Biotechnology), anti-phosphoPKA substrates (rabbit polyclonal, Cell Signaling), anti-GAPDH (mouse monoclonal, Santa Cruz Biotechnology), and anti-tubulin (mouse monoclonal, Sigma-Aldrich). Secondary antibodies were purchased from Amersham Life Sciences (GE Healthcare). Bands were visualized by enhanced chemiluminescence (ECL; Amersham Life Sciences) according to the manufacturer’s instructions, and were quantified by using densitometry (Chemidoc, Bio-Rad). Each experiment and densitometric quantification was separately repeated at least three times.

### RNA Isolation and Real-Time PCR

Total RNA was prepared using TRIzol (Invitrogen, Eugene, OR, United States), according to the manufacturer’s instruction. Oligo-dT first strand cDNA was synthesized using the SuperScript VILO cDNA Synthesis Kit (Invitrogen, Life technologies) according to the manufacturer’s instructions. mRNA expression was determined in cardiac samples from different experimental groups by real-time quantitative PCR (RT-PCR) using a IQ-5 Multicolor Real-Time PCR Detection System (BIORAD). The primers used were: β-MHC: forward 5′-GAGACGACTGTGGCCTCC-3′, reverse 5′-GCATGATGGCGCCTGTCAG-3′; Collagen IA1: forward 5′-GGAGACAGGTCAGACCTGTGTG-3′, reverse 5′-CAGCTGGATAGCGACATCGGC-3′; Collagen III: forward 5′-ATATCAAACACGCAAGGC-3′, reverse 5′-GATTAAAGCAAGAGGAACAC-3′; Fibronectin: forward 5′- ACCGTGTCAGGCTTCCGG-3′, reverse 5′- ACGGAAGTGGCCGTGCTT-3′; and GAPDH: forward 5′-TGCAGTGGCAAAGTGGAGATT-3′, reverse 5′-TCGCTCCTGGAAGATGGTGAT-3′.

### Mitochondria Isolation and Evaluation of Respiratory Parameters

Mitochondria were isolated from cardiac samples and respiration rate was detected on isolated mitochondria as previously described (Perrino et al., 2013; Schiattarella et al., 2016). Briefly, hearts were gently homogenized in a buffer containing 220 mM mannitol, 70 mM sucrose, 20 mM Tris⋅HCl, 1 mM EDTA, and 5 mM EGTA (pH 7.4) and then centrifuged at 8,000 *g* for 10 min at 4°C. The supernatant was further centrifuged and the mitochondrial pellet was either use for immunoblot analysis or to measure mitochondrial respiration (Perrino et al., 2013; Schiattarella et al., 2016).

### Adult Ventricular Murine Myocytes Isolation

Adult ventricular myocytes were isolated from murine adult hearts using a modified heart retrograde perfusion-based method. Briefly, mice were injected with heparin and anesthetized with an intraperitoneal injection of 0.1 ml/kg of mixture of 50% Tiletamine and 50% Zolazepam (Zoletil 100) and Xylazine 5 mg/kg (Sigma-Aldrich). The heart was quickly excised, and the aorta was cannulated for retrograde perfusion in a Langendorff apparatus at a constant flow rate of 1.5 ml/min at 37°C. The heart was perfused for 9–10 min with isolation buffer [NaCl 120 mM, KCl 4.4 mM, MgCl_2_ 1 M, NaH_2_PO_4_ 1.2 mM, NaHCO_3_ 20 mM, glucose 5 mM, 2,3-butanedione monoxime (BDM) 1.25 mM, and taurine 5 mM, pH 7.4], bubbling the isolation solution with 95% O_2_ – 5% CO_2_, followed by digestion for 13 min with collagenase II (350 U/ml; Worthington) in isolation buffer. After digestion, myocytes were suspended in isolation buffer, filtered with a mesh (100 μm), gently spin down (300 g for 1 min), and resuspended for stepwise Ca^2+^ reintroduction from 25 μmol/L to 1.0 mmol/L. Myocytes were than lyzed in a buffer containing Tris 1 M, 1% v/v Nonidet P-40, NaCl 5 M, 10% w/v deoxycholate, NaVO_3_ 100 mM, and NaF 100 mM to extract protein as described above.

### Histology

Mouse heart specimens were fixed in 4% formaldehyde and embedded in paraffin. After de-paraffinization and re-hydratation, 4-μm-thick sections were prepared and mounted on glass slides. An even number of cardiac cross sections per group were stained with wheat germ agglutinin (WGA) or Picosirius red as previously described (Schiattarella et al., 2016). Briefly, thin cardiac sections were analyzed using Nikon light microscope and NIS Elements Basic Research software (Nikon). For assessment of cardiomyocyte cross sectional area, mean area was evaluated by measuring 400–500 cells per heart (*n* = 4–5 animals/group). Fibrotic regions (6–8 fields/section, *n* = 4–5 animals/group) were measured as percent of collagen-stained area/total myocardial area and averaged using a computer-assisted image analysis software (Image J software, National Institutes of Health).

### TUNEL Staining

Cardiac DNA nicks were assayed by an *in situ* Apoptosis Detection kit or ApopTag Fluorescein Direct *in situ* Apoptosis Detection kit (Chemicon) according to manufacturer’s instructions as previously described (Esposito et al., 2015; Schiattarella et al., 2016). TUNEL staining was visualized by specific green fluorescence and nuclei by 4′-6-diamidino-2-phenylindole (DAPI). TUNEL-positive cardiomyocytes nuclei identified were normalized by total nuclei stained in the same sections by DAPI (*n* = 7–8 animals/group). An even numbers of slides were analyzed for each group.

### Statistical Analysis

All data presented are representative of three or more independent experiments and are expressed as mean ± SEM. Comparisons between two groups were performed using the unpaired Student’s *t*-test. For experiments including three or more experimental groups, comparisons were made by one-way analysis of variance (ANOVA) or 2-way ANOVA, and *p* values shown indicate the effect of genotype response. Correction for multiple comparisons was made using the Student–Newman–Keuls method. A minimum value of *p* < 0.05 was considered statistically significant. All statistical analyses were conducted with Prism statistical software.

## Results

### *Akap1* Deletion Enhances Cardiac Hypertrophy After Transverse Aortic Constriction in Mice

**FIGURE 1:**
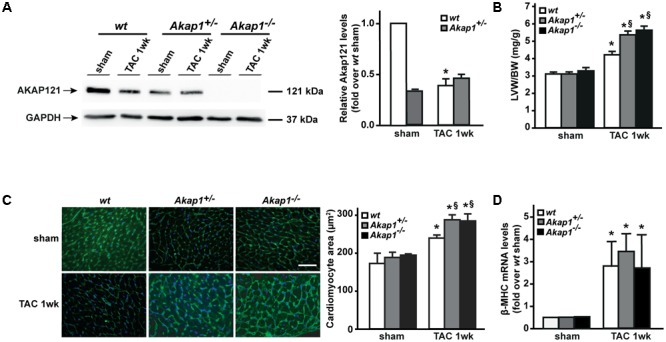
*Akap1* deficiency exacerbates pressure overload-induced cardiac hypertrophy. **(A)** Representative immunoblot (left) and densitometric analysis (right) of AKAP121 protein levels in hearts from sham (*n* = 5) and 1 week transverse aortic constriction (TAC 1w; *n* = 7) operated *wt, Akap1*^+/-^, and *Akap1*^-/-^ mice. GAPDH was used as control of loading sample (^∗^*p* < 0.05 vs. *wt* sham). **(B)** Bar graphs showing cumulative data of left ventricular weight (LVW) to body weight (BW) 1 week after sham or TAC procedures in *wt, Akap1*^+/-^, and *Akap1*^-/-^ mice (^∗^*p* < 0.05 vs. sham; ^§^*p* < 0.05 vs. *wt* TAC 1w; *n* = 7 hearts/group). **(C)** (Left) Representative images of wheat germ agglutinin (WGA) staining of cardiac transversal sections from *wt, Akap1*^+/-^, and *Akap1*^-/-^ mice 1 week after sham or TAC procedure. DAPI was used as nuclei counterstaining. Scale bar: 20 μm. (Right) Bar graphs showing cumulative data of multiple independent experiments analyzing cardiomyocytes cross-sectional area (^∗^*p* < 0.05 vs. sham; ^§^*p* < 0.05 vs. *wt* TAC 1w; *n* = 5 hearts/group). **(D)** mRNA levels of β-myosin heavy chain (β-MHC) in cardiac samples from *wt, Akap1*^+/-^, and *Akap1*^-/-^ mice 1 week after sham or TAC procedure (^∗^*p* < 0.05 vs. sham; *n* = 5 hearts/group).

**FIGURE 2:**
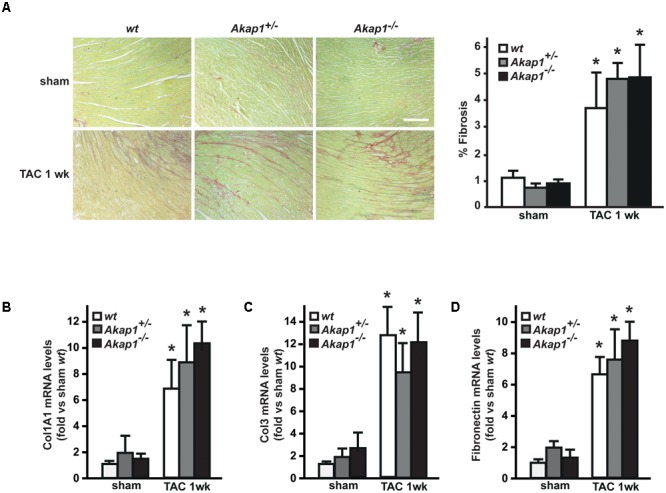
Loss of *Akap1* does not affect cardiac fibrosis response after pressure overload. **(A)** (Left) Representative images of Picrosirius red staining of cardiac transversal sections from *wt, Akap1*^+/-^, and *Akap1*^-/-^ mice 1 week after sham or TAC procedure. Scale bar: 40 μm. (Right) Bar graphs showing cumulative data of multiple independent experiments analyzing percentage of fibrosis (^∗^*p* < 0.05 vs. sham *wt*; *n* = 5 hearts/group). mRNA levels of collagen I (Col1A1, **B**), collagen III (Col3; **C**), and fibronectin **(D)** in cardiac samples from *wt, Akap1*^+/-^, and *Akap1*^-/-^ mice 1 week after sham or TAC procedure (^∗^*p* < 0.05 vs. sham; *n* = 5 hearts/group).

We have previously demonstrated that myocardial levels of AKAP121 decrease in a rat model of LVH induced by ascending aortic banding (Perrino et al., 2010), suggesting a role for this adaptor protein in the transmission of hypertrophic signals in the myocardium. However, the effects of *Akap1* genetic deletion on cardiac remodeling in response to pressure overload are currently unknown. Consistent with our previous results, AKAP121 cardiac levels were significantly decreased in *wt* mice subjected to 1w TAC compared to sham-operated littermates (**Figure 1A**), and these results were associated with the impairment of mitochondrial PKA signaling (Supplementary Figure S1). Importantly, AKAP121 downregulation was also observed in adult ventricular myocytes isolated from *wt* TAC hearts (Supplementary Figure S2), demonstrating that AKAP121 downregulation in LVH occurs in cardiomyocytes. Interestingly, after TAC, both *Akap1*^+/-^ and *Akap1*^-/-^ mice exhibited a significant further increase in LVW/BW compared to *wt* TAC mice (**Figure 1B** and **Table 1**), coupled with an increase in cardiomyocytes cross-sectional area measured by WGA staining (**Figure 1C**) and in β-myosin heavy chain (β-MHC) mRNA abundance (**Figure 1D**). No differences were found among the three different genotypes in cardiac interstitial fibrosis, measured by Picrosirius red staining (**Figure 2A**), as well as in the mRNA abundance of the most common fibrotic markers: collagen type I, type III, and fibronectin (**Figures 2B–D**). Collectively, these data suggest that loss of *Akap1* exacerbates cardiac hypertrophy induced by pressure overload without affecting the fibrotic response.

**Table 1:** Echocardiographic and morphometric analysis in the different experimental groups of mice.

	*wt*		*Akap1*^+/-^		*Akap1*^-/-^	
	Sham (*n* = 8)	TAC 1w (*n* = 12)	Sham (*n* = 6)	TAC 1w (*n* = 13)	Sham (*n* = 6)	TAC 1w (*n* = 9)
**Morphometry**					
BW (g)	27.3 ± 1.4	23.0 ± 0.7*	23.3 ± 0.8	23.6 ± 0.9	23.4 ± 1.1	23.0 ± 0.5
LVW (mg)	84.9 ± 4.9	100.4 ± 5.9*	73.6 ± 3.3	124.0 ± 4.4*§	76.9 ± 7.0	99.6 ± 5.3*#
HW (mg)	112.5 ± 7.0	131.8 ± 7.8	95.8 ± 4.7	154.6 ± 4.7*§	105.8 ± 10.2	135.1 ± 7.3*#
LVW/BW (mg/g)	3.1 ± 0.1	4.2 ± 0.2*	3.1 ± 0.1	5.4 ± 0.2*§	3.3 ± 0.2	5.7 ± 0.6*§
HW/BW (mg/g)	4.9 ± 0.1	5.6 ± 0.3*	4.1 ± 0.1	6.6 ± 0.2*	4.5 ± 0.3	5.9 ± 0.3*
**Echocardiography**					
LVEDd (mm)	3.0 ± 0.1	3.1 ± 0.1	3.0 ± 0.1	4.0 ± 0.1*§	3.2 ± 0.1	3.6 ± 0.1*§
LVESd (mm)	1.2 ± 0.1	1.6 ± 0.1*	1.2 ± 0.1	2.7 ± 0.1*§	1.3 ± 0.1	2.4 ± 0.2*§
IVS,d (mm)	0.7 ± 0.0	1.1 ± 0.0*	0.8 ± 0.0	1.0 ± 0.0*	0.8 ± 0.0	1.0 ± 0.0*
PW,d (mm)	0.8 ± 0.0	0.9 ± 0.0*	0.8 ± 0.0	1.0 ± 0.0*	0.8 ± 0.0	0.9 ± 0.1
FS (%)	60.6 ± 1.8	50.3 ± 2.3*	60.0 ± 1.2	32.5 ± 2.8*§	58.6 ± 2.3	33.5 ± 3.4*§
HR (bpm)	573 ± 11	583 ± 17	595 ± 30	584 ± 23	681 ± 19	661 ± 16

*BW, body weight; LVW, left ventricle weight; HW, heart weight; LVEDd, left ventricular end-diastolic diameter; LVESd, left ventricular end-systolic diameter; IVS,d interventricular septum end-diastolic diameter; PW,d posterior wall end-diastolic diameter; FS, fractional shortening; HR, heart rate (^∗^*p* < 0.05 vs. correspondent sham; **^§^***p* < 0.05 vs. *wt* TAC 1w; and ^#^*p* < 0.05 vs. *Akap1*^+/-^ TAC 1w).*

### Genetic Deletion of *Akap1* Precipitates Heart Failure Induced by Pressure Overload

**FIGURE 3:**
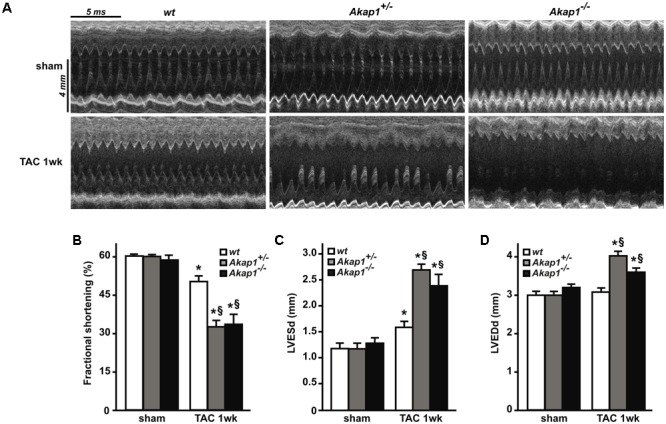
Genetic deletion of *Akap1* accelerates the progression toward heart failure in TAC mice **(A)**. Representative left ventricular (LV) M-mode echocardiographic tracings from *wt, Akap1*^+/-^ and *Akap1*^-/-^ mice after sham or TAC 1w procedures (*n* = 7 mice/group). Cumulative data of % fractional shortening **(B)**, left ventricular end-systolic diameter (LVESd; **C**) and left ventricular end-diastolic diameter (LVEDd; **D**) in *wt, Akap1*^+/-^ and *Akap1*^-/-^ mice 1 week after sham or TAC procedure (^∗^*p* < 0.05 vs. sham; ^§^*p* < 0.05 vs. TAC 1w *wt*; *n* = 5 hearts/group).

Chronic pressure overload inevitably leads to impairment in LV systolic function and ultimately HF (Esposito et al., 2011; Schiattarella and Hill, 2015). As expected, after 1w TAC, *wt* mice exhibited a mild but significant reduction in % FS compared to sham-operated animals (**Figures 3A,B** and **Table 1**). Reduction in % FS in *wt* mice was mainly due to the increase in LV end-systolic diameter (LVESd, **Figure 3C**) without significant changes in LV end-diastolic diameter (LVEDd, **Figure 3D**). In *Akap1*^+/-^ and *Akap1*^-/-^ mice, TAC operation caused a significant further decrease of % FS compared to TAC *wt* mice (**Figures 3A,B**). Reduction in % FS observed in *Akap1*^+/-^ and *Akap1*^-/-^ after TAC, resulted by increase in both LVESd and LVEDd (**Figures 3C,D**). The differences in LV systolic function between *wt* and *Akap1*-deficient mice after stress suggest that *Akap1* plays an important role in the progression toward HF after chronic pressure overload.

### Increased Apoptosis and Blunted *Akt* Activation in *Akap1*^-^*^/^*^-^ Hearts After Pressure Overload

**FIGURE 4:**
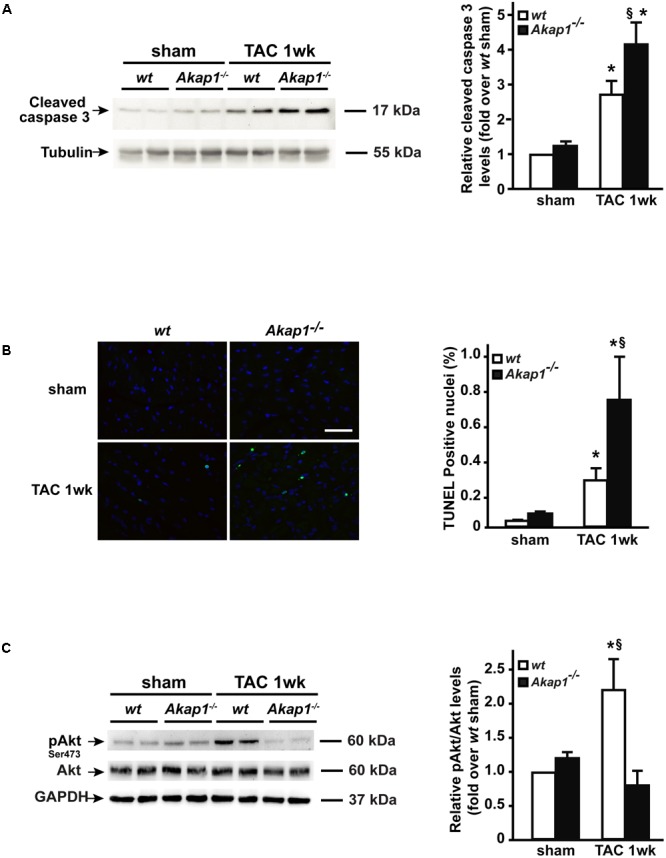
Increased cardiac apoptosis and lack of Akt activation in *Akap1*^-/-^ mice after TAC. **(A)** Representative immunoblot (left) and densitometric analysis (right) of cleaved caspase-3 protein levels in *wt* and *Akap1*^-/-^ hearts 1 week after sham or TAC procedure (^∗^*p* < 0.05 vs. sham; ^§^*p* < 0.05 vs. *wt* TAC 1w; *n* = 5 hearts/group). Tubulin was used as control of loading sample. **(B)** (Left) Representative DAPI and TUNEL staining of cardiac sections from *wt, Akap1*^+/-^, and *Akap1*^-/-^ mice 1 week after sham or TAC procedure. Positive nuclei appear green. Scale bar: 20 μm. (Right) Bar graphs showing cumulative data of multiple independent experiments evaluating TUNEL-positive cells (^∗^*p* < 0.05 vs. sham; ^§^*p* < 0.05 vs. *wt* TAC 1w; *n* = 5 hearts/group). **(C)** Representative immunoblot (left) and densitometric analysis (right) of Akt Ser 473 phosphorylation (pAkt) and Akt in *wt* and *Akap1*^-/-^ hearts 1 week after sham or TAC procedure. GAPDH was used as control of loading sample (^∗^*p* < 0.05 vs. sham; ^§^*p* < 0.05 vs. *Akap1*^-/-^ TAC 1w; *n* = 5 hearts/group).

Reduction in LV systolic function is dependent on loss of contractile cellular elements, since cardiomyocytes apoptosis occurs in the context of pressure overload stress. Thus, we next investigated the presence of apoptosis in *wt* and *Akap1*^-/-^ TAC hearts. Consistently with cardiac functional data presented above, myocardial levels of cleaved caspase-3, a well-known effector of apoptosis, increased after 1w of TAC in *wt* mice (**Figure 4A**). Strikingly, in *Akap1*^-/-^ hearts, we observed a significant further increase in cleaved caspase-3 myocardial levels compared to *wt* animals (**Figure 4A**). To confirm that increased apoptosis was present in *Akap1*^-/-^ hearts, we quantified the number of TUNEL-positive nuclei in myocardial sections from *Akap1*^-/-^ and *wt* mice. Consistent with previous data, after TAC, the % number of TUNEL-positive cells was significantly higher in *Akap1*^-/-^ hearts compared to *wt* littermates (**Figure 4B**). In order to determine the contribution of mitochondrial-dependent apoptotic pathways in the absence of *Akap1*, we evaluated the levels of the mitochondrial apoptotic protein caspase-9 in cardiac lysates from *wt* and *Akap1*^-/-^ mice. Interestingly, *Akap1*^-/-^ hearts exhibited a significant increase in caspase-9 cleavage compared to *wt* hearts after both sham and TAC procedure (Supplementary Figure S3). These data indicate that loss of *Akap1*^-/-^ increased the susceptibility to pressure overload-induced apoptosis, which is, at least in part, dependent by activation of mitochondrial pro-apoptotic pathways.

Activation of Akt-dependent protective signals is a hallmark of HF (Chaanine and Hajjar, 2011). Recently, we have shown that loss of *Akap1* in vascular endothelial cells (ECs) blunts ischemia-induced Akt activation resulting in dysfunctional behavior of ECs (Schiattarella et al., 2018). Given the fact the *Akap1*^-/-^ mice have an accelerated progression toward HF after pressure overload and that *Akap1*^-/-^ hearts are more susceptible to stress-induced apoptosis, we hypothesized that loss of cardioprotective signals from Akt might play a role in this context. Activation of Akt (Ser473 phosphorylation) occurred in *wt* hearts after TAC (**Figure 4C**), whereas *Akap1*^-/-^ hearts exhibited a blunted activation of Akt in response to pressure overload (**Figure 4C**). The absence of Akt activation together with the enhanced apoptotic rate observed in *Akap1*^-/-^ hearts suggests that both mechanisms might act in concert to determine the impaired cardiac function of *Akap1*^-/-^ mice after pressure overload.

### *Siah2* Deletion Prevents AKAP121 Degradation After Pressure Overload

**FIGURE 5:**
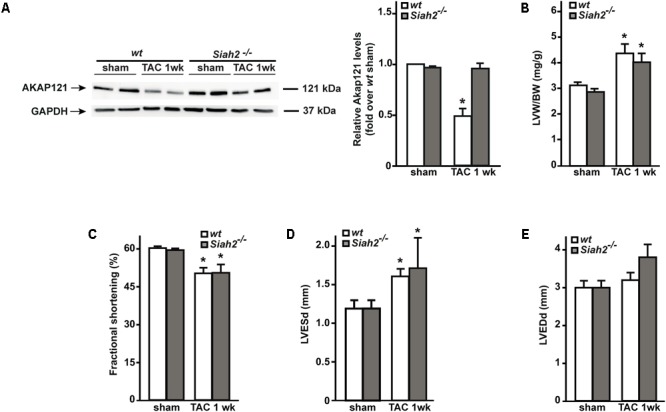
Siah2 deficiency prevents AKAP121 degradation after TAC. **(A)** Representative immunoblot (left) and densitometric analysis (right) of AKAP121 protein levels in *wt* and *Siah2*^-/-^ hearts 1 week after sham or TAC procedure. GAPDH was used as control of loading sample (^∗^*p* < 0.05 vs. *wt* sham). **(B)** Bar graphs showing cumulative data of LVW/ BW 1 week after sham or TAC procedures in *wt* and *Siah2*^-/-^ mice (^∗^*p* < 0.05 vs. sham; *n* = 6 hearts/group). Cumulative data of % fractional shortening **(C)**, left ventricular end-systolic diameter (LVESd; **D**), and left ventricular end-diastolic diameter (LVEDd; **E**) in *wt* and *Siah2*^-/-^ mice 1 week after sham or TAC procedure (^∗^*p* < 0.05 vs. sham; *n* = 6 hearts/group).

Others and we have previously demonstrated that the absence of *Siah2* prevents hypoxia-induced AKAP121 degradation (Schiattarella et al., 2016; Kim et al., 2011). To investigate whether *Siah2* deletion might prevent AKAP121 degradation in response to pressure overload, *Siah2*^-/-^ mice underwent the TAC procedure. One week after surgery, downregulation of AKAP121 cardiac levels was significantly inhibited in *Siah2*^-/-^ mice compared to *wt* littermates (**Figure 5A**). Interestingly, *Siah2*^-/-^ mice exhibited the same extent of cardiac hypertrophy estimated by LVW/BW compared to *wt* controls (**Figure 5B**), as well as similar deterioration of cardiac function investigated by means of FS% (**Figure 5C**), LVESd (**Figure 5D**), and LVEDd (**Figure 5E**).

## Discussion

In the present study, we demonstrate for the first time that *in vivo* genetic deletion of *Akap1* exacerbates pressure overload-induced cardiac hypertrophy development and accelerates the progression toward HF after TAC in mice. These abnormalities in *Akap1*^-/-^ mice are associated to an increased rate of cardiac apoptosis and lack of activation of Akt-dependent cardioprotective signals. These results extend the knowledge about *Akap1* in cardiac biology, and confirm its role as a critical mediator of pathological LVH.

Cardiac hypertrophy is the first, general response of the heart to physiological or pathological loads (Chien, 1999; Frey and Olson, 2003). However, while cardiac adaptations induced by physiological stimuli such as exercise do not result in HF, cardiac overload induced by pathological stimuli, such as chronic hypertension or heart valve disease, eventually leads to cardiac dysfunction (Berenji et al., 2005; Heineke and Molkentin, 2006; Perrino et al., 2006). Abnormalities in cAMP/PKA signaling are a hallmark of pathological LVH and HF (Perrino and Rockman, 2007). AKAPs scaffold proteins compartmentalize PKA activity into subcellular domains (e.g., mitochondria), thus allowing a tight-controlled spatial and temporal regulation of cellular responses (Feliciello et al., 2001; Newhall et al., 2006).

The transmission of cAMP signaling to mitochondria is reached by a family of mitoAKAPs that have been shown to play an important role in cardiac response to stress (Perrino et al., 2010; Diviani et al., 2011). In particular, others and we have shown that AKAP121 is critical for cardiomyocytes response to ischemic injury (Kim et al., 2011; Schiattarella et al., 2016). Genetic deletion of *Akap1* results in larger infarct size, worse LV systolic function, and increased mortality after myocardial infarction (Schiattarella et al., 2016). Although the absence of *Akap1* was associated to increased mitochondrial dysfunction, mitophagy, and ROS production, the specific mechanisms by which loss of *Akap1* results in worse post-ischemic cardiac remodeling remain elusive. More recently, we have shown that AKAP121 also regulates ECs behavior in response to ischemia through Akt signaling (Schiattarella et al., 2018). In ECs, lack of *Akap1* affects multiple cellular functions and results in reduced Akt activation upon angiogenic stimuli or hypoxia (Schiattarella et al., 2018). In the present investigation, we demonstrated that in response to pressure overload, deletion of *Akap1* results in exacerbated LVH in the absence of Akt activation, confirming in a different experimental model of cardiovascular disease that the presence of AKAP121 is necessary to achieve Akt activation in response to stress.

A previous study has shown that AKAP121 acts as a repressor of cardiomyocytes hypertrophy (Abrenica et al., 2009). Silencing of AKAP121 *in vitro* resulted in increased cardiomyocytes cell size in the absence of any pro-hypertrophic stimulus (Abrenica et al., 2009). Although our data confirm the role of AKAP121 as a brake of cardiomyocytes growth, others and we repetitively observed the absence of any basal cardiac alterations in *Akap1*^-/-^ mice (Newhall et al., 2006; Schiattarella et al., 2016, 2018). In-depth characterization of cardiac structure and function in *Akap1*^-/-^ mice revealed that absence of *Akap1* did not cause LVH and HF over time. In addition, we have previously demonstrated that *in vivo* administration of synthetic peptides displacing AKAP121 from mitochondria increased cardiac ROS and apoptotic cell death, but is was not sufficient to induce cardiac hypertrophy, even if we observed increased nuclear localization of nuclear factor of activated T-cells (NFAT), the main effector of the calcineurin-dependent pro-hypertrophic signaling pathway (Perrino et al., 2010). However, when subjected to pressure overload, *Akap1*-deficient mice develop a more robust hypertrophic response and an accelerated progression toward HF, confirming that *Akap1* acts as a repressor of cardiomyocytes hypertrophy. The fact that, only if stressed, *Akap1*^-/-^ hearts exhibit pronounced cardiac hypertrophy might be in contrast with the previous notion that knockdown of AKAP121 *in vitro* affects *per se* cardiomyocyte size. However, this apparent discrepancy can be explained by the fact that, *in vivo*, Rab32, a mitochondria-targeted AKAP-like protein, might exert compensatory effects in the absence of *Akap1* (Alto et al., 2002; Bui et al., 2010). The pivotal role of AKAP121 in the regulation of HF development was also confirmed by the evidence that while *Akap1*-deficient mice underwent rapid deterioration of cardiac function in response to pressure overload, and this process was not observed in *Siah2*-deficient mice.

The absence of *Akap1* has been variously associated with increased ROS production and mitochondrial dysfunction in several models of cardio- and cerebrovascular diseases as well as in cancer (Feliciello et al., 1998; Scorziello et al., 2013; Schiattarella et al., 2016; Rinaldi et al., 2017). For example, despite no basal differences were found in mitochondrial respiration between *Akap1*^-/-^ and *wt* hearts, following myocardial infarction, *Akap1*^-/-^ mice exhibited increase levels of cardiac ROS and more prominent alterations in mitochondrial morphology compared to *wt* controls (Schiattarella et al., 2016). After 1w-TAC, no differences were observed between *Akap1*^-/-^ and *wt* mice in cardiac mitochondrial respiratory function (Supplementary Figure S4). However, we cannot exclude that alterations in mitochondrial function might occur at a later time point in *Akap1*^-/-^ hearts in the context of pressure overload. These results suggest that mitochondrial morphological and functional alterations induced by the absence of *Akap1* might be influenced by the nature and/or duration of stress that triggers cardiac dysfunction.

As mentioned above, the absence of *Akap1* leads to lack of Akt activation in hypertrophic hearts, and we speculate that Akt could mediate the effects of *Akap1* deletion on hypertrophic growth. Akt is at the crossroad of signaling pathways regulating cardiac growth and contractile function (Aoyagi and Matsui, 2011; Chaanine and Hajjar, 2011). Being downstream of insulin/insulin-like growth factor (IGF), Akt activation has been associated to physiological LVH development (Ellison et al., 2012). However, Akt phosphorylation has also been found increased in pathological LVH as an initial response to afterload stress (Chaanine and Hajjar, 2011). Therefore, Akt activation in TAC has been recognized as a pro-survival adaptation signal, required for the activation of protein synthesis in cardiomyocytes (Matsui et al., 1999; Fujio et al., 2000; Cannavo et al., 2013). Consistent with this notion, *Akap1*^-^*^/^*^-^ mice subjected to TAC exhibited increased cardiac cell death and an accelerated progression toward HF coupled with lack of Akt activation. A number of signaling molecules are placed downstream of Akt. Among these, the mechanistic target of rapamycin (mTOR) plays a central role in cardiomyocyte hypertrophy (Morales et al., 2016). Recently, *Akap1* has been shown to control mTOR regulating cancer cells growth (Rinaldi et al., 2017). However, the mechanisms by which *Akap1* influences Akt/mTOR signaling pathway remain poorly understood. Akt localizes to diverse sub-cellular compartments, including mitochondria, where it contributes to phosphorylation of key mitochondrial targets (Bijur and Jope, 2003; Miyamoto et al., 2008; Lim et al., 2016). The absence of *Akap1* leads to reduction in Akt phosphorylation in LVH as well as in vascular dysfunction (Schiattarella et al., 2018), suggesting that mitoAKAPs might also contribute to Akt subcellular distribution to promote cell survival. Hence, it is possible to hypothesize that mitoAKAPs might act as a signaling platform contributing to stabilize Akt mitochondrial localization to preserve mitochondrial function and promote survival. Further studies will be necessary to clarify the precise mechanism(s) by which loss of *Akap1* prevents Akt activation upon different stressors.

## Conclusion

Our findings support the critical role of mitoAKAPs in cardiac responses to pressure overload, identifying Akt as potential mediator of their anti-hypertrophic effects.

## Author Contributions

GS, NB, RP, FC, VT, AFr, SD’A, GG, LR, DB, and AG performed the experiments and made the analyses. GS and CP wrote the manuscript. AL, AFe, and GE contributed to the experimental design and manuscript preparation. CP conceived the project.

## Conflict of Interest Statement

The authors declare that the research was conducted in the absence of any commercial or financial relationships that could be construed as a potential conflict of interest.
