# Supplementary material for: Loss of Akap1 Exacerbates Pressure Overload-Induced Cardiac Hypertrophy and Heart Failure
Source: Front Physiol. 2018 May 28;9:558. doi: 10.3389/fphys.2018.00558 (PMC5985454; doi:10.3389/fphys.2018.00558)
Supplement: Supplementary file 1 [file Presentation_1.PDF]

Supplementary Figure 1

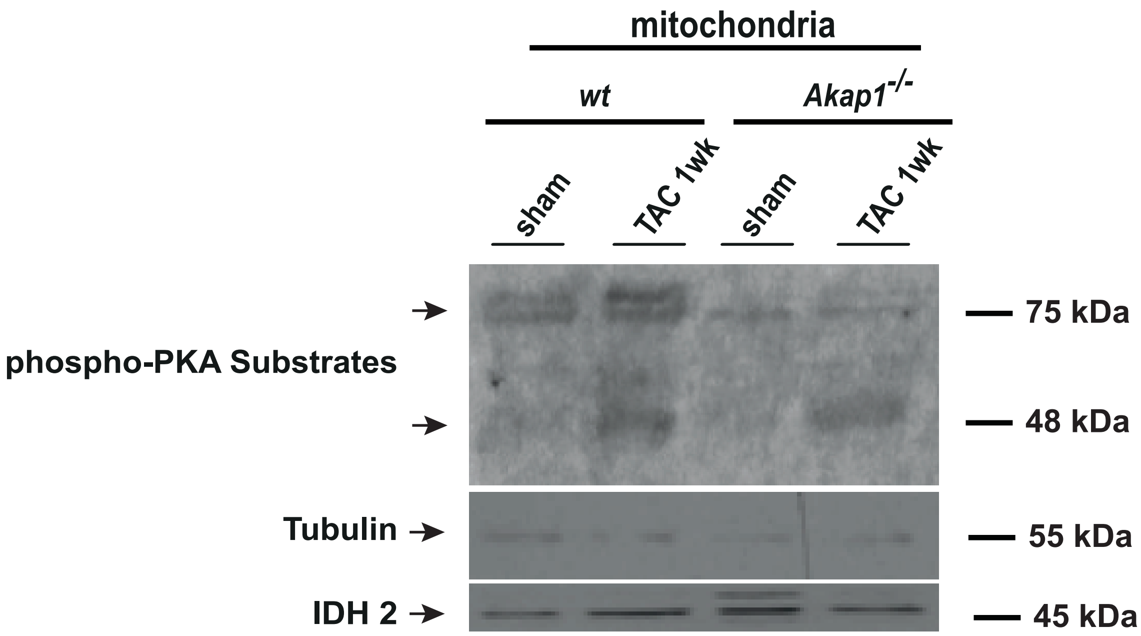

Supplementary Figure 1

Phospho-PKA substrates in mitochondrial cardiac samples from *wt* and *Akap1*$^{-/-}$ mice after sham or TAC procedure
Tubulin (cytosolic) and IDH2 (mitochondrial) proteins were used as control of subcellular fractionation.

Supplementary Figure 2

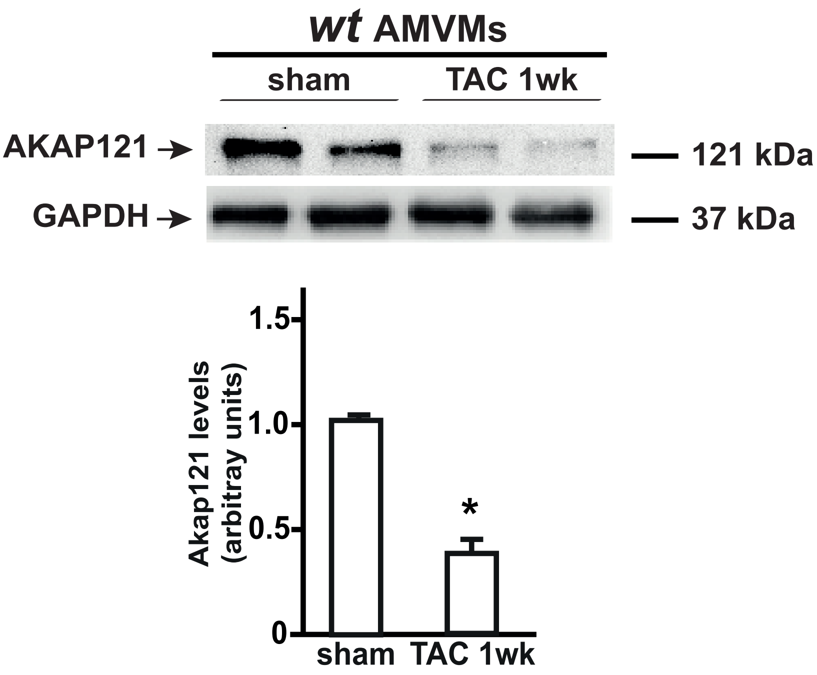

Supplementary Figure 2

AKAP121 protein levels in cardiomyocytes isolated from wild-type TAC mice
Representative immunoblot (top) and densitometric analysis (bottom) of three independent experiments to evaluate AKAP121 protein levels in cardiomyocytes (adult mouse ventricular myocytes, AMVMs) from *wt* sham or TAC hearts one week after the procedure (*$p<0.05$ vs. sham).
GAPDH was used as control of loading sample.

Supplementary Figure 3

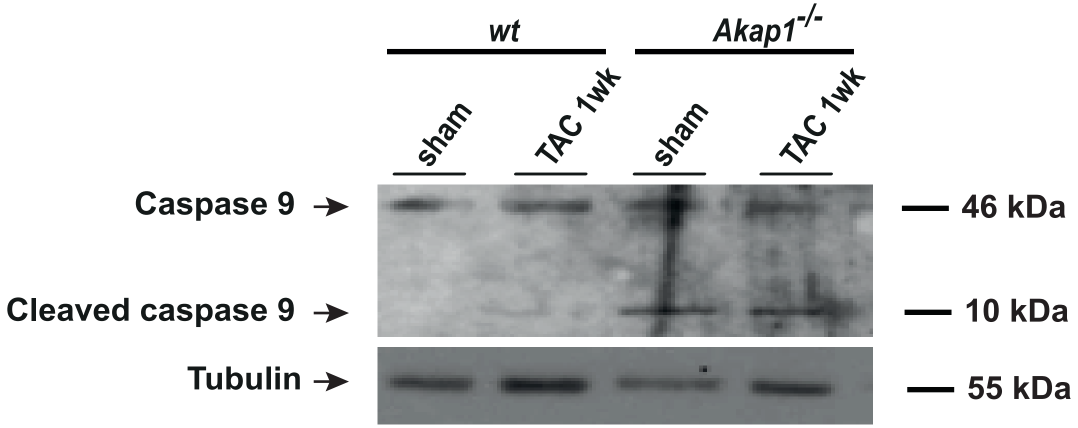

Supplementary Figure 3

Caspase 9 activation in cardiac samples from *wt* and *Akap1*$^{-/-}$ mice after sham or TAC procedure
Tubulin protein was used as loading control.

Supplementary Figure 4

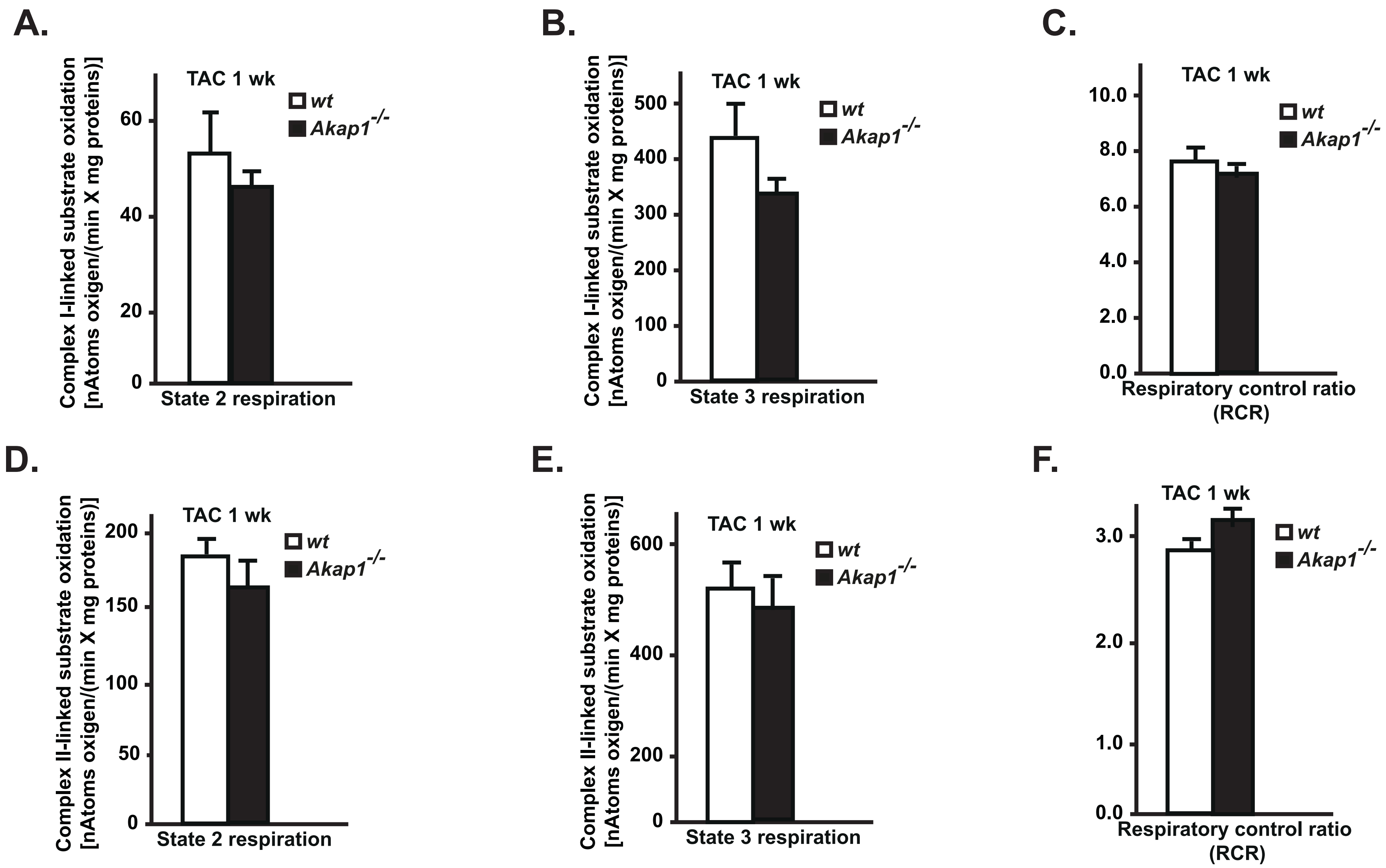

Supplementary Figure 4

Absence of differences in mitochondrial respiration between *wt* and *Akap1*$^{-/-}$ hypertrophic hearts

Mitochondrial respiratory parameters [State 2, State 3 and respiratory control ratio (RCR)] in *wt* and *Akap1*$^{-/-}$ hearts after one week of TAC. Parameters were detected in presence of complex I-linked substrate (Piruvate+malate) (A-C) and complex II-linked substrate (succinate+rotenone) (D-E). Bar graphs show mean ± s.e.m. of 4 hearts/group. Each experiment was performed in duplicate.
